# The role of bacteriological monitoring using culture and drug susceptibility tests (CDST) on treatment outcomes among MDR/RR-TB patients on treatment: a cohort analysis of patients enrolled on treatment 2010-2015 in Zimbabwe

**DOI:** 10.11604/pamj.2021.39.97.26796

**Published:** 2021-06-02

**Authors:** Ronnie Matambo, Shungu Mutero-Munyati, Vongai Mildred Pepuka, Tendai Nkomo, Charles Sandy, Mkhokheli Ngwenya, Gilchriste Ndongwe, Elliot Chikaka, Sungano Mharakurwa, George Nyandoro

**Affiliations:** 1 Biomedical Research and Training Institute, 10 Seagrave Ave, Harare, Zimbabwe,; 2 Ministry of Health and Child Care, AIDS and TB, Harare, Zimbabwe,; 3 World Health Organisation, Country Office, Harare, Zimbabwe,; 4 Zimbabwe Evidence Informed Policy Network, Harare, Zimbabwe,; 5 Africa University, Faculty of Health Agriculture and Natural Resources, Mutare, Zimbabwe,; 6 AIDS and TB Department, Ministry of Health and Child Care, Harare, Zimbabwe

**Keywords:** Multi-drug resistant tuberculosis, bacteriological monitoring, drug susceptibility tests, drug resistant tuberculosis, tuberculosis treatment outcomes

## Abstract

**Introduction:**

an estimated 25% of the world population is infected with Mycobacterium tuberculosis. In 2017, new tuberculosis cases were estimated at 10 million, while 1.6 million tuberculosis related deaths were recorded, 25% residing in Africa. Treatment outcomes of multi drug resistant Tuberculosis patients in Zimbabwe has been well documented but the role of bacteriological monitoring on treatment outcomes has not been systematically evaluated. The objective of the study was to determine the role of bacteriological monitoring using culture and drug susceptibility tests on treatment outcomes among patients with multi drug resistant tuberculosis.

**Methods:**

a retrospective, secondary data analysis was conducted using routinely collected data of patients with multi drug resistant TB in Zimbabwe. Frequencies were used to summarize categorical variables and a generalized linear model with a log-link function and a Poisson distribution was used to assess factors associated with unfavourable outcomes. The level of significance was set at P-Value<0.05.

**Results:**

about the study collected data from 473 records of patients with an average age of 36.35 years. Forty-nine percent (49%) were male and 51% were female. Results showed that when a patient has baseline culture result missing, has no culture conversion result, regardless of having a follow up culture and drug susceptibility test result, the risk of developing unfavourable outcomes increase by 3.9 times compared to a patient who has received all the three (3) bacteriological monitoring tests.

**Conclusion:**

results highlights the need for consistent bacteriological monitoring of patients to avert unfavourable treatment outcomes.

## Introduction

The world has an estimated population of 25% infected with Mycobacterium tuberculosis (MTB), the bacteria that causes tuberculosis (TB). In 2017, there were an estimated 10 million incident TB patients globally and 1.6 million deaths due to TB disease of which 25% resided in Africa [1]. In addition in the same year, 558,000 new cases of MDR-TB were estimated, but only 161,000 were detected and reported, while 139,000 were initiated on second line treatment for multidrug-resistant tuberculosis (MDR-TB) [1]. Of the top 30 most TB burdened countries in the world, twelve Southern African countries are included [1]. Improving access to health care especially in impoverished TB-stricken regions of the world is as important as conducting molecular surveillance in the control of MTB [1]. MDR/RR-TB treatment outcomes have been found to be directly associated with programmatic characteristics such as delay in treatment initiation, duration of treatment, type of drug sensitivity testing, individualized treatment regimens and use of directly observed therapy [2,3]. Fueled by socio-economic migration in the region, multi-drug resistance and rifampicin resistant TB (MDR/RR-TB) poses a major threat to TB care in low to middle income countries (LMIC). WHO reports that only 55% of patients with MDR/RR-TB are successfully treated [4]. In order to successfully control MDR/RR-TB, early detection, timely initiation of treatment and thorough monitoring of treatment outcomes forms the basis for care [5]. Mostly in LMIC, poor and mostly nonfunctional health care systems tend to negate gains in implementation of such care. Furthermore, the development of extensively drug resistant TB (XDR-TB) normally results from poor care provided to patients with MDR/RR-TB.

Zimbabwe is among the 14 high burden countries (HBCs) with a triple burden of TB, TB/HIV and MDR-TB [6]. In 2017 Zimbabwe had an estimated 37,000 incident TB patients and 8,300 TB-associated deaths. In the same report, an estimated 1,300 MDR/RR-TB patients in the country in the same year with a prevalence of 4.6% and 14% among new and previously treated TB patients respectively was reported [6]. The national TB programme (NTP) of Zimbabwe released the “programmatic management of drug resistant TB” (PMDT) guidelines in 2010 with use of standardized second line drugs (SLDs) for twenty months. The World Health Organisation (WHO) estimated that less than 40% of the MDR/RR-TB patients were diagnosed and put on treatment in Zimbabwe in 2017 [7]. In the same report, among those initiated on treatment, more than 50% had unfavourable treatment outcomes. There has been no systematic assessment on the role of bacteriological monitoring using culture and drug susceptibility tests (CDST) on treatment outcomes among patients on MDR/RR-TB treatment under the Zimbabwe NTP nor has there been assessment of the individual and programmatic factors associated with treatment outcomes. A study by the same author R. Matambo *et al*. on-treatment outcomes of MDR/RR-TB patients in Zimbabwe reported a high proportion of missing CDST results among patients enrolled into the routine programme [8]. The impact of missing CDST results on treatment outcomes was however not ascertained. This is cause for concern as patients must be managed based on their bacteriological status to improve their treatment outcomes. We therefore conducted a follow up study aimed at assessing the role of bacteriological monitoring using CDST on treatment outcomes among patients initiated on MDR/RR-TB treatment under the Zimbabwe national TB programme (NTP) between 2010 and 2015. The study therefore aims to report the role of bacteriological monitoring using CDST on treatment outcomes among patients on MDR/RR-TB treatment.

## Methods

**Study design:** this was a retrospective cohort study using Individual data routinely collected within the Zimbabwe National TB Programme.

**General setting:** Zimbabwe has an estimated population of 17 million people and is landlocked. It shares borders with Mozambique to the east, South Africa to the South, Zambia to the North and Botswana to the South West [9]. The country is divided into ten provinces, two of which are Metropolitan provinces (Harare, the capital city and Bulawayo, the second largest city). The provinces are further sub divided into 62 districts. The country´s public healthcare referral system constitutes four levels: 1) the quaternary level constituting six central hospitals located in the two metropolitan provinces, 2) the tertiary level consisting eight provincial hospitals which are the highest referral hospitals providing selected basic medical specialties for the eight rural provinces, 3) the secondary level constituting at least one district and or general hospital per district and last 4) the primary care level consisting of rural and urban healthcare facilities that provide primary health care services.

**Diagnosis of MDR/RR-TB:** in Zimbabwe, TB diagnosis and treatment services are provided in public healthcare facilities and are integrated with general health services. Private laboratories however complement the efforts by also providing TB diagnosis services for private patients. Prior to 2013, only previously treated sputum positive pulmonary TB patients and MDR-TB contacts were considered as presumptive MDR-TB patients and evaluated for MDR-TB. Their sputum specimens were subjected to either phenotypic (culture and drug susceptibility testing (CDST) or genotypic (MTB/Rif assay) testing. From 2013 onwards, Xpert MTB/Rif assay was used upfront for diagnosis of TB and rifampicin resistance in MDR-TB high risk groups (retreatment TB, chest symptomatic of MDR-TB contacts, those HIV-positive, health workers with pulmonary TB (PTB), miners with PTB and children <5 years). In all Rifampicin resistant (RR-TB) patients, the remainder of the two collected sputum specimens is sent to one of the country´s two national reference laboratories for CDST in order to assess drug susceptibility to all the first line drugs.

**Treatment initiation and follow-up MDR/RR-TB:** all diagnosed MDR/RR-TB patients are registered and started on treatment at either district or provincial hospitals or at polyclinics and infectious disease hospitals in metropolitan provinces. The district medical officer is responsible for providing oversight on the clinical management of all MDR/RR-TB patients in their respective districts. On registration at district hospital, the patient is notified to the national TB programme (NTP) and a patient-held drug resistant TB (DR-TB) treatment card is issued. The treatment card is updated simultaneously with the health facility directly observed treatment (DOT) DR-TB register at all patient follow-up visits by health facility workers. The health facility DOT DR-TB register also documents socio-demographic and clinical details of the MDR/RR-TB patients. Patient data in the health facility DOT register are also entered into the DR-TB register which is maintained and updated by DR-TB co-ordinators. As part of pre-treatment evaluation for all patients, laboratory investigations such as liver function tests, renal function tests and complete blood count are supposed to be done prior to initiation of treatment. The district clinical management team initiates treatment based on pre-treatment evaluation. Details of the clinical and laboratory examinations are documented in the DR-TB card and in the clinical notes attached to the DR-TB card. During the study period, the WHO recommended standardised DOTS-Plus regimen be used for management of MDR/RR-TB patients [9]. The duration of treatment was at least 20 months with a minimum of six months (and 4 months after culture conversion) in the intensive phase and 14 months of the continuation phase. Oral drugs namely levofloxacin, pyrazinamide, cycloserine and ethambutol were given both during the intensive and continuation phases. The injectable kanamycin was provided six days a week during intensive phase. Treatment dosages were dispensed based on patient weight.

After treatment initiation, patients are monitored for two weeks at facilities where treatment was initiated before considering if the patient is stable and tolerating the regimen. Those considered “stable” were patients who were able to ingest medication, did not show signs of adverse drug reaction and had all the laboratory investigations within normal limit. Based on clinical severity and distance of travel from the patient´s residence (10 km), they are either admitted and monitored or asked to visit the district hospital daily. After two weeks, based on proximity to a health facility, patients either continue DOTS-plus in district hospitals or they are referred to primary health facilities nearest to their residence. Patients are followed-up as per PMDT guidelines and the programmatic treatment outcomes are ascertained by the medical officer. If a patient developed side-effects due to kanamycin, their dosage was reduced however currently they are switched to capreomycin [9]. Patients are also offered provider-initiated HIV testing services in the MDR-TB pre-treatment phase and those found to be HIV positive are assessed for initiation on antiretroviral therapy (ART) and cotrimoxazole preventive therapy (CPT). As per the national guidelines in use during the study period, they were initiated on a fixed-dose combination once-daily pill of Tenofovir v+ Lamuvidine (or Emtricitabine) + Efavirenz (TDF+3TC) or (FTC)+EFV) as the preferred first-line ART regimen among adult PLHIV and abacavir+ lamuvidine + efavirenz or (Lopinavir/r) (ABC + 3TC + EFV (or Lop/r)) as the preferred first-line ART regimen in children living with HIV [10].

**Bacteriological monitoring:** during the study period 2010-2015, patients were monitored according to the programmatic management of drug resistant TB in Zimbabwe. This entailed collection of sputum sample monthly for CDST throughout the treatment duration.

**Study population:** all MDR/RR-TB patients initiated on treatment between 2010 and 2015 under the Zimbabwe NTP were included in the study and clinical details of the patients who had a CDST done at baseline, had CDST to ascertain culture conversion and at any other period before completion of treatment (follow up CDST) were documented and analysed.

**Data variables, sources of data and data collection:** patient demographic and clinical data were extracted from the health facility DOT register, individual patient clinical notes and the district DR-TB register using a structured proforma. All data were subjected to quality assurance at source of collection wherein 10% of the records were randomly verified by the district health environmental officer of the respective districts. Data were extracted during August to December 2018.

**Data entry and analysis:** data were double entered and validated using EpiData entry software (EpiData association, Odense, Denmark) and data were analysed using Stata version 15. Frequencies and percentages were used to describe all categorical variables and age variable was described using the mean and median (IQR). Frequencies were used to summarize categorical variables and generalized linear modelling with a log-link function and a Poisson distribution was used to assess adjusted factors associated with unfavourable outcomes. The level of significance was set as P-Value <0.05.

## Results

**Demographics:** the study enrolled 473 participants shown in Table 1. These were confirmed MDR/RR-TB patients. Most patients were between 25 to 34 years, 169 (35.7%) followed by 35 to 44 years, was 34 years with an interquartile range between 29 to 42 years. The National TB programme initiated a total of 935 MDR/RR 149 (31.5%). The median age was 34 years with an interquartile range between 29 to 42 years. The National TB programme initiated a total of 935 MDR/RR-TB patients on treatment during the study reference period of which 473 (51%) were followed up at district hospitals and urban healthcare facilities and were included in the study. The 473 patients in our study contributed 672.5 person-years of follow-up time.

**Table 1:** demographic characteristics of MDR/RR-TB patients initiated on treatment during 2010 to 2015 in Zimbabwe

Characteristic (n =473)		n (%)
**Sex**	Male	230 (48.6)
	Female	241 (51.0)
	Missing	2 (<1)
**Age**	<24	68 (14.4)
	25-34	169 (35.7)
	35-44	149 (31.5)
	45-54	47 (9.9)
	55+	34 (7.2)
	Not recorded	6 (1.3)
	Median (IQR)	34 (29 - 42)
**Marital status**	Married	202 (42.7)
	Single	143 (30.2)
	Widowed	44 (9.3)
	Divorced	25 (5.3)
	Missing	59 (12.5)

IQR = interquartile range

Table 2 shows that HIV status was a significant predictor for unfavourable MDR/RR-TB outcomes at univariate level. HIV positive (RR=1.57, p value = 0.010) or Untested patients with unknown HIV status (RR=2.24, p value = 0.044) was associated with high risk of developing unfavourable treatment outcomes, < 0.005. Table 3 shows frequencies and results for tests on bacteriological monitoring associated effect in terms of risk imposed towards developing unfavourable outcomes p value < 0.005. Table 3 shows frequencies and results for tests on bacteriological monitoring associated effect in terms of risk imposed towards developing unfavourable outcomes, the respective p values for significance testing. The main questions that were asked during data recording among MDR-TB patients were: (a) was baseline culture and drug susceptibility test done? (b) does the patient have a culture conversion result? (c) does the patient have a follow up CDST result after first culture conversion result?

**Table 2:** clinical characteristics against unfavourable MDR-TB outcomes among patients initiated on MDR/RR-TB treatment in Zimbabwe

Clinical characteristics		Frequency, n (%)	Unadjusted risk ratio (RR)	P-value
*HIV status	*Positive	352(74.4%)	1.57 (1.11 - 2.22)	0.010
	Negative	101(21.4%)	base	-
	*Unknown/untested	5(1.1%)	2.24 (1.02 - 4.93)	0.044
	Missing	15(3.2%)	1.50 (0.74 - 3.01)	0.259
**Type of TB**	New	257(54.3%)	base	-
	Retreatment after loss to follow-up	19(4.0%)	0.92 (0.50 - 1.69)	0.786
	Retreatment after failure	116(24.5%)	0.92 (0.70 - 1.22)	0.586
	Relapse	81(17.1%)	0.95 (0.70 - 1.31)	0.774
**Baseline sputum microscope examination**	Yes	343(72.5%)	base	-
	No		1.12 (0.84 - 1.48)	0.437
	Missing		1.14 (0.78 - 1.66)	0.495
**Initial culture examination done**	Yes	301 (63.64%)	base	-
	No	157(33.19%)	1.19 (0.94 - 1.50)	0.153
	Missing	15 (3.17%)	1.09 (0.58 - 2.07)	0.781
*Month of culture conversion	1 month	11 (2.33%)		
	2 months	24 (5.07%)	1.83 (0.46 - 7.26)	0.388
	3 months	77 (16.28%)	1.00 (0.26 - 3.83)	1.000
	4 months	69 (14.59%)	1.28 (0.34 - 4.81)	0.719
	5 months	44 (9.30%)	1.25 (0.32 - 4.91)	0.749
	6 months	34 (7.19%)	1.13 (0.27 - 4.68)	0.864
	7 months	7 (1.48%)	1.57 (0.28 - 8.75)	0.606
	8 months	2 (0.42%)	-	-
	9 months	12 (2.54%)	1.38 (0.28 - 6.76)	0.695
	10 months	2 (0.42%)	-	-
	11 months	3 (0.63%)	1.83 (0.24 - 14.03)	0.559
	12 months	2 (0.42%)	-	-
	*Missing	186 (39.32%)	3.58 (1.02 - 12.61)	0.047
*Sputum microscope examination at 5months?	Yes	300 (63.42%)	Base	-
	*No	142 (30.02%)	2.58 (2.03 - 3.26)	<0.001
	*Missing	31 (6.55%)	2.92 (2.16 - 3.94)	<0.001

**Table 3:** univariate analysis of bacteriological monitoring effect on MDR-TB treatment outcomes

Bacteriological monitoring test	Frequency, n (%)	Unadjusted risk Ratio (RR)	P- value
Baseline culture (yes), culture conversion result (yes), follow up CDST (yes)	118 (24.95%)	base	-
Baseline culture (yes), culture conversion result (yes), follow up CDST (missing)	66 (13.95%)	1.17 (0.65 - 2.08)	0.602
Baseline culture (yes), culture conversion result (no), follow up CDST (missing)	53 (11.21%)	2.90 (1.88 - 4.49)	<0.001
Baseline culture (missing), culture conversion result (no) follow up CDST (yes)	48 (10.15%)	3.95 (2.66 - 5.89)	<0.001
Baseline culture (missing), culture conversion result (yes) follow up CDST (yes)	37 (7.82%)	1.53 (0.82 - 2.83)	0.180
Baseline culture (missing), culture conversion result (no) follow up CDST (yes)	21 (4.44%)	2.93 (1.74 - 4.94)	<0.001
Baseline culture (yes), culture conversion result (no), follow up CDST (yes)	64 (13.53%)	3.37 (2.24 - 5.06)	<0.001
Baseline culture (missing), culture conversion result (yes) follow up CDST (missing)	66 (13.95%)	1.09 (0.60 - 1.97)	0.780

Favourable treatment outcomes included a) Treatment completed, b) cured, while unfavourable outcome included a) Treatment failure, b) Died, c) Lost to follow up, d) not evaluated Results of the univariate analysis binomial regression modelling show unadjusted risk ratios and respective frequency tabulation summary. The order of risk towards developing unfavourable outcomes is as follows: (a) 1st observed scenario - the highest risk (RR=3.95, p value < 0.001) of developing unfavourable outcome was among patients without a baseline CDST result, had no culture conversion tests result, although the patient had a follow up culture and drug susceptibility test result available (CDST) at any period during treatment. (b) 2^nd^ observed scenario - the 2^nd^ highest risk (RR=3.37, p value < 0.001) of developing unfavourable outcome was among patients with a baseline CDST result, had no culture conversion result but had follow up CDST result. (c) 3^rd^ observed scenario - when a patient had a missing baseline CDST result, had no culture conversion result, but had follow up CDST result, the risk of developing unfavourable MDR-TB outcomes was higher (RR=2.93, p value < 0.001). (d) 4^th^ observed scenario is when a patient had baseline culture results, had no culture conversion result and had missing CDST follow up results, the risk of developing unfavourable MDR-TB outcomes was (RR=2.90, p value < 0.001).

Table 4 below show adjusted risks hence risk categories by order of magnitude of the relative risk (RR) values: 1^st^ higher risk category (RR=2.77, p value < 0.001) was when a patient had baseline CDST done, had no culture conversion result but had follow up CDST done at some point during treatment. 2^nd^ risk category was when a patient had only a baseline CDST done, had no culture conversion result and had no follow up CDST done at any point during treatment. Here there was a significantly high risk (RR=2.22, p value <0.001) of developing unfavorable MDR-TB treatment outcomes. 3^rd^ risk category was when a patient did not have baseline CDST, had no culture conversion result and had no follow up CDST done, RR 206, p value=0.013. 4^th^ risk category had patients who had a sample collected for sputum microscopy but had a missing or undocumented result for sputum microscopy examination at 5 months, RR=2.0, p value <0.001. 5^th^ risk category were patients who did not have a sputum microscopy examination done at 5 months RR=1.83, p value <0.001.

**Table 4:** adjusted risks categories by order of magnitude of the relative risk (RR) values

	Outcome category	RR	95% conf interval	p-value
	Sex	1.00	0.820-1.220	0.987
	Age	1.01	1.000-1.010	0.094
*monitoring	Baseline culture (Yes), culture conversion result (yes), follow up CDST (no)	1.14	0.640-2.000	0.660
	*Baseline culture (yes), culture conversion result (No), follow up CDST (no)	2.22	1.430-3.450	0.000
	Baseline culture (No), culture conversion result [No], Follow up CDST (no)	1.89	0.960-3.720	0.066
	Baseline culture (No), culture conversion result (Yes), follow up CDST (yes)	0.97	0.410-2.290	0.936
	Baseline culture (No), culture conversion result [No], follow up CDST (yes)	1.61	0.790-3.280	0.188
	*Baseline culture (Yes), culture conversion result (No), follow-up CDST (yes)	2.77	1.830-4.180	0.000
	Baseline culture (No), culture conversion result (yes), follow-up CDST(no)	0.70	0.310-1.600	0.402
	Baseline culture (no), culture conversion result (no), follow up CDST (no)	2.06	1.16-3.640	0.013
Baseline sputum microscopy	No	0.90	0.710-1.150	0.392
	Missing	1.02	0.740-1.420	0.896
*Follow up sputum microscopy at 5 months	*No	1.83	1.440-2.330	0.000
	*Missing	2.00	1.430-2.780	0.000
Baseline culture	No	1.58	0.890-2.810	0.116
	Missing	1.00		
	_cons	0.14	0.080-0.220	0.000

## Discussion

**Statement of principle findings** one out of four patients (25%) had all the 3 bacteriological monitoring results available and these patients developed favourable treatment outcomes. When classified according to risk, patients who did not have any of the monitoring results were in the third risk category. These are patients who did not have baseline culture, had no culture conversion result and had no follow up drug susceptibility test, RR 206, p value=0.013.

**Strengths and weaknesses of the study:** this was the first nationwide study to assess the role of bacteriological monitoring using CDST on treatment outcomes among MDR/RR-TB patients enrolled in a national progamme. The study however could not proffer reasons for the high proportion of missing CSDT results.

**Strengths and weaknesses in relation to other studies:** since there is no known study that specifically focused on the role of bacteriological monitoring using CDST on treatment outcomes of MDR/RR-TB patients, we could not evaluate the strengths and weaknesses of our study in relation to other studies thus our study in the absence of known literature on the same topic, was a ground breaking study.

**Discussion in important differences in results:** we could not discuss important differences with other studies as there are no known studies that specifically focused on the same or similar topic with our study.

**Meaning of the study:** the study findings show that bacteriological monitoring of MDR/RR-TB patients using CDST improves treatment outcomes. Conversely, failure to bacteriologically monitor MDR/RR-TB patients on treatment increases their risk of an unfavourable outcome.

**Unanswered questions and future research:** since this was a retrospective study, we could not determine whether the high proportion of missing CDST results were due to i) specimen not collected, ii) specimen collected but result contaminated, iii) Results not relayed back to facility. Knowledge of exact cause would have helped proffer relevant recommendations to the NTP. We thus recommend future studies to inform the possible gaps

It is not known why most patients lacked access to baseline CDSTs before treatment was commenced. Knowledge of the reasons will allow the NTP to close this gap in future thus affording patients enrolled into the programme proper assessment of appropriateness of treatment as results become available. Future research should be conducted on ways to strengthen sputum collection transportation and testing the NTP would need to know why delivery of results back to requesting facilities was such a challenge so as to put in appropriate measures in ensuring adherence to national guidelines on sputum collection, transportation and delivery of results. The NTP would need to explore if modern technologies such as a short message services (SMSs) which has improved result turn-around times in early infant diagnosis as compared to courier-based reporting would improve the challenges in the programme [10]. This may ultimately improve treatment outcomes as patients receive results on time. The NTP should also investigate if investment at hub level in point-of-care diagnostics that offer culture and drug sensitivity patterns reduce the proportion of missing results due to accessibility. This initiative proved useful in other programmes [11]. The NTP should make all consented efforts to find out if the further the testing laboratory is, the more the risk of specimen and results leakages.

The NTP should also in future consider implementing operational research on an Electronic tracking system which technology tracks specimens referred and results delivered. This minimises losing specimens along the delivery chain and leakages of results. Lack of such a system was reported as a barrier to accessing SL-DSTs in India [12]. During the study period, the economic situation prevailing in Zimbabwe presented challenges in transporting the paper-based laboratory results to health facilities due to fuel shortages. The question therefore the NTP should answer is how resilient the public health system is in Zimbabwe? A study by Murongazvombo *et al*. [13] reported failure to collect sputum specimens by the clinicians for CDST, even when sputum specimens were collected, some did not reach the reference laboratory owing to various reasons. Future studies should be conducted to inform these reasons. Even when specimens reached the Reference laboratory, specimen leakages may have stemmed from culture no growths and culture contaminations as described in a study by Timire *et al*. [14]. All these questions not answered undermine the critical role of bacteriological monitoring and may impact treatment outcomes. As this study has shown, patients who did not receive adequate bacteriological monitoring ended up with unfavourable treatment outcomes. The NTP would need to document all challenges with processes of sputum specimen transportation to National Reference laboratories as these have been reported in other countries by Kilale *et al*. [15].

**Study limitations:** first, as this was a retrospective study, we could not determine whether the high proportion of missing CDST results were due to i) specimen not collected, ii) specimen collected but result contaminated, iii) results not relayed back to facility. Knowledge of exact cause would have helped proffer relevant recommendations to the NTP. Second, the patients who were referred to rural primary healthcare facilities for follow-up care after MDR-TB treatment initiation were not included. Compared to patients referred, the patients included in our study were more likely coming from areas closer to district hospitals with access to healthcare services. Thus, the current study cohort was more likely to have had higher proportion of samples collected and transported to national reference laboratories and results delivered to them as compared to the cohort that was excluded due to distance and thus the later cohort were more likely to have a comparable worse treatment outcomes.

## Conclusion

Failure to have bacteriological monitoring result as specified in the PMDT guidelines in use during the study period prejudiced some patients of a favourable treatment outcome. The NTP should ensure access to CDST for all patients and Strengthen specimen referral and result feedback.

### What is known about this topic

- High proportion of missing CDST results in patients under MDR/RR-TB care;
- Patient management is predominantly clinical rather than bacteriological;
- Most patients on MDR/RR-TB Treatment experience unfavourable treatment outcomes as compared to Patients on drug susceptible TB regimen.

### What this study adds

- Consistent bacteriological monitoring of patients using CDST improves treatment outcomes of MDR/RR-TB patients on treatment;
- Improves risk perception in clinicians on the impact of offering in adequate care to patients.
